# Intelligence

**Published:** 1827-11

**Authors:** 


### INTELLIGENCE.

### MONTHLY REPORT OF PREVALENT DISEASES.

The damp close weather of autumn has given rise to many cases of fever, with hepatic affections. The latter have been so prevalent, that we have occasionally seen two or three new cases of jaundice in a day, at a public institution which we attend. We have likewise seen two cases apparently hepatic, which are of considerable interest: one is in the person of a young woman, who at first sight presents the appearance of being pregnant; on examination, a tumor can be felt descending from under the ribs on both sides, and extending half-way between the umbilicus and pubes; here it terminates, and the fingers can be as it were turned under the edge of a resisting body, which is supposed to be the liver. There is but little fever, and no considerable inconvenience beyond the weight and bulk of the tumor, which has been ten months in acquiring its present dimensions. In the other case, the tumor occupies the epigastric region, extending also into the right hypochondrium: it is easily perceptible to the eye, feels irregular on the surface, and gives the idea of being twice the size of an orange. The subject is a boy about ten years of age, who is much out of health, having hectic fever, with expectoration of blood mixed with purulent matter. It is three years since the tumor was first perceived.

Rheumatism, in a rather severe form, has also been prevalent during the last month, and we continue to find calomel and opium, in full doses, the most efficient method of treatment whenever the disease becomes acute.

*Medico-Chirurgical Society.*—This Society, under the presidentship of Mr. Travers, aided by the exertions of the Secretaries, has assumed a degree of vigour which it has not enjoyed, till now, since the first years of its establishment. The meetings commenced on Tuesday, October 9th, and the opening of the season was attended by many of the most eminent members of the profession; on which occasion a paper on Erysipelas, by Mr. Lawrence, was read. Mr. Brodie, Mr. Travers, Dr. Gregory, Dr. Johnson, Dr. A. T. Thomson, were among the speakers; but, as the transactions are hereafter to appear at short intervals, we shall abstain from noticing the papers till they come regularly before us.

*Westminster Medical Society.*—On Saturday evening, October 20th, this Society resumed its meetings for the season. The attendance was very numerous; but, from the injudicious custom of choosing office-bearers on the first evening, the time which ought to have been devoted to discussion was occupied in conversation, and writing ballotting lists. This ought to be reformed: either a separate night ought to be set apart for business of this nature, or the meeting ought to be called an hour earlier. At length the time for *professional* business having arrived, Dr. Milligan related some cases of Enlargement of the Spleen in children after remittent fever, which had yielded to Iodine; mercury having been, in the first place, freely employed without success.

Mr. Mayo afterwards related a curious case of Fracture and Dislocation of the Spine, in which a man, falling from a height, had struck his back against a beam. Mr. M. found the patient with the body, as it were, doubled up, the lower part of the spine being projected behind the upper, at the ninth lumbar vertebra, to some extent. The upper part of the body was fixed, and the lower extremities pulled down with a gentle but continued extension, and the spinal column thus brought as nearly into its natural situation as could be effected without violence. The patient experienced considerable relief, and became *sensible of having lower extremities*, which he had not been before. The parts below the fracture were paralysed, and the urine required to be drawn off by the catheter. The accident occurred five days ago, yet the patient appeared to have no symptoms which threatened immediate danger, and, what was rather remarkable, the urine had not become ammoniacal. Active means, especially in the way of local depletion, had been employed.

A discussion then followed on similar cases, in which Mr. Mayo, Mr. Rose, Dr. Barry, Dr. Johnson, Mr. Lambert, and others, took part. The principal point on which difference of opinion existed was, whether the ammoniacal condition of urine in such cases depended on a diseased state of the secretion, or on its being retained in the bladder till spontaneous decomposition commenced. This latter opinion was maintained by Mr. Dillon, who asserted that in hospitals the urine was sometimes allowed to remain twenty-four or thirty-six hours in the bladder; but he did not inform the Society in what hospitals this took place.

Mr. Rose showed the absurdity of attributing the evils resulting from severe injury to the spine, to the secondary effect of paralysing the bladder, and denied the general assertion with regard to neglect in drawing off the urine when such cases were treated in public hospitals. He alluded to the general fact that injuries of the back, in a very large proportion of cases, give rise to an ammoniacal state of the urine; and stated that it possesses this character when it is not suffered to remain in the bladder at all, but is withdrawn by the catheter immediately as it flowed from the kidneys; showing the change to depend on the impaired function of these glands.

Dr. Barry remarked, that as, according to the experiments of the French physiologists, the division of the eighth pair of nerves prevents the formation of acid in the stomach, so the injury to the spine might, on a similar principle prevent the formation of acid in the kidneys, and thus all ow the secretion to assume an alkaline character.

*Medico-Botanical Society.*—THE first meeting of the eighth session of the Medico-Botanical Society of London was holden on Friday evening, the 12th of October, 1827, at the Society's apartments, 32, Sackville-street, Piccadilly, Sir JAMES M'GRIGOR, president, in the chair.

The Minutes of the last meeting having been read, several presents to the Society were announced, amongst which were some of the seeds of the Argemone Mexicana, a mild purgative, and of the Genista Tinctoria, a plant used by the Russians as a cure for hydrophobia.

The director (Mr. FROST) delivered the Annual Oration, which he commenced by showing the advantages derivable from the extended sphere of the Society, and its use to the medical officers of the army and navy; he then pointed out the salutary effects that would accrue from the regulations relative to the study of botany by them, instituted by the director-general of the Army Medical Board.

A notice was read, offering a reward of 25*l.*, or a gold medal of equal value, from an accurate description of the plant yielding the Myrrh, and which is merely supposed to be the produce of the Amyris Kataf.

After some remarks from the president, assuring the members of the constant interest he took in the welfare of the Society, and pointing out the steps he had taken, and would take, for the promotion of its objects, the meeting was adjourned to Friday evening, the 9th of November.

The room, which was crowded to excess, was decorated with a numerous collection of shrubs and flowers, amongst which were the Sago, the Tan and Date Palms, the Tea-tree, the Akee-tree, Ficus Religiosa, Mimosa Sensitiva, a new species of Cassia-Laurius Benzoin, &c. &c.

---

*New Regulations of the Company of Apothecaries.*—WE inserted in our last Number a copy of these Regulations, and we recur to the subject for the purpose of directing the attention of pupils, and their parents or guardians, to them. Nothing is more certain than that the education of the general practitioner has been very much improved since the establishment of the Apothecaries' Company on its present plan, and we cannot but commend the laudable desire evinced by that body to render it still more perfect.

The principal changes in the present regulations relate to the order of succession in which the various branches of medicine shall be cultivated; and when we state that they are almost identical with those recommended by us in this Journal for May 1825, it will scarcely be necessary to add that they meet with our humble approbation. At the time alluded to, we ventured to dissent from the order of study recommended by Dr. PARIS, in the Introduction to his Elements of Medical Chemistry, and added, "we would be disposed to recommend anatomy, chemistry, and materia medica, as the rudiments of medical science, to be assiduously and exclusively studied during the first year; and the theory and practice of medicine and surgery during the second, when the student would be much better able to appreciate them."

Viewing the matter in the same light, the Apothecaries have resolved that candidates for their certificates shall have attended Lectures on Materia Medica, Chemistry, and Anatomy, (one course of each,) before his attendance on the Practice of Medicine; while his attendance at a hospital or dispensary must commence after one course of these last lectures.

These regulations will oblige pupils to be in London two seasons, and, although we perceive that an attempt has elsewhere been made to excite dissatisfaction among the pupils by representing this as a hardship, we certainly must say that the measure appears to us calculated to raise the scale of medical acquirement throughout England, and that whatever inconvenience the pupils, or those entrusted with their education, may experience in the first instance, will be much more than compensated to the young practitioner by his increased respectability, and by the consciousness of being competent to meet the exigencies of his arduous profession in a manner the most advantageous to his patient, and most conducive to his own comfort, credit, and reputation.

---

*Amputation of the Jaw.*—We are informed that Mr. HODGSON, of Birmingham, has recently performed the operation of amputating the greater portion of the lower jaw, in a case of osteo-sarcoma of that bone. The result has been most satisfactory, the patient having completely recovered in three weeks.

---

*Action against the Lancet.*—MANY persons having applied to us verbally, and some by letter, on the subject of our proceedings against the Lancet, we beg to inform all who may feel any interest in the matter, that we neither have, nor ever have had, any intention of abandoning our action. The insinuation to the contrary is "a weak invention of the enemy," in which there is as little truth as in the statement on which the prosecution itself is grounded.

### METEOROLOGICAL JOURNAL,

*From September 20th, to October 20th,* 1827.

By Messrs. HARRIS and Co. Mathematical Instrument Makers, 50, High Holborn.

| September | Rain gauge. | Moon. | Thermom. 9 A.M. | Thermom. MAX. | Thermom. MIN. | Barometer. 9 A.M. | Barometer. 10P.M. | De Luc's Hygrom. 9 A.M. | De Luc's Hygrom. 10P.M. | Winds. 9 A.M. | Winds. 10P.M. | Atmospheric Variations. 9 a.m. | Atmospheric Variations. 2 p.m. | Atmospheric Variations. 10 p.m. |
|---|---|---|---|---|---|---|---|---|---|---|---|---|---|---|
| 20 | .28 | | 48 | 55 | 49 | 29.65 | 29.80 | 91 | 88 | S | ENE | Rain | Cloudy | Cloudy |
| 21 | | ● | 51 | 64 | 54 | 29.71 | 29.55 | 90 | 90 | W | WSW | Fair | — | Sl. Rain |
| 22 | | | 55 | 64 | 49 | 29.46 | 29.40 | 90 | 85 | W | WSW | Overca. | — | Fine |
| 23 | .48 | | 54 | 61 | 50 | 29.40 | 29.52 | 89 | 86 | WSW | SW | Sl. Rain | Rain | — |
| 24 | | | 51 | 62 | 52 | 29.54 | 29.61 | 91 | 85 | SW | SW | Fine | Fair | Fair |
| 25 | | | 54 | 64 | 53 | 29.59 | 29.55 | 90 | 88 | SW | S | Overca. | Fine | — |
| 26 | .32 | | 58 | 69 | 57 | 29.49 | 29.47 | 95 | 99 | S | SE | — | Overca. | Rain |
| 27 | | | 60 | 66 | 56 | 29.46 | 29.61 | 99 | 93 | SE | SSW | Fair | Fair | Cloudy |
| 28 | | ☽ | 59 | 64 | 54 | 29.60 | 29.63 | 94 | 98 | S | S | Cloudy | — | — |
| 29 | .46 | | 58 | 61 | 55 | 29.53 | 29.59 | 95 | 95 | SE | S | — | Rain | — |
| 30 | | | 57 | 64 | 57 | 29.65 | 29.66 | 78 | 87 | SW | SE | — | Fair | Fair |
| Oct. | | | | | | | | | | | | | | |
| 1 | | | 58 | 64 | 53 | 29 67 | 29.68 | 96 | 93 | SSE | SE | Overca. | Cloudy | Rain |
| 2 | | | 54 | 63 | 52 | 29.77 | 29.96 | 100 | 93 | SSE | SE | — | Fair | Fine |
| 3 | | | 57 | 61 | 51 | 30.09 | 30.18 | 95 | 91 | ENE | NNE | Cloudy | — | — |
| 4 | | | 52 | 63 | 51 | 30.24 | 30.27 | 96 | 95 | NNE | NE | Foggy | — | — |
| 5 | | ○ | 55 | 65 | 49 | 30.27 | 30.18 | 100 | 92 | ENE | NNE | Cloudy | — | — |
| 6 | | | 54 | 64 | 47 | 30.05 | 29.95 | 95 | 94 | ESE | SE | Fair | — | — |
| 7 | | | 49 | 58 | 48 | 29.83 | 29.71 | 95 | 95 | SE | SE | Foggy | Foggy | — |
| 8 | | | 54 | 62 | 53 | 29.54 | 29.34 | 92 | 95 | S | SSW | Fair | Fair | Rain |
| 9 | .57 | | 55 | 59 | 51 | 29.21 | 29.16 | 98 | 95 | SSW | WSW | Rain | Rain | — |
| 10 | .68 | | 53 | 58 | 47 | 29.17 | 29.10 | 95 | 98 | WSW | E | Cloudy | — | — |
| 11 | .7 | | 50 | 54 | 48 | 29.13 | 29.15 | 90 | 88 | W | WSW | Fair | — | Fair |
| 12 | | | 51 | 57 | 43 | 29.24 | 29.43 | 93 | 86 | WNW | W | — | Fair | — |
| 13 | | | 43 | 46 | 44 | 29.40 | 29.44 | 89 | 89 | W | W | — | — | — |
| 14 | | ☾ | 48 | 57 | 51 | 29.56 | 29.69 | 86 | 81 | W | W | — | — | — |
| 15 | | | 55 | 63 | 57 | 29.81 | 29.83 | 95 | 96 | W | SW | Overca. | Cloudy | Cloudy |
| 16 | | | 60 | 64 | 52 | 29.83 | 29.83 | 96 | 95 | WSW | SW | — | Fair | Fair |
| 17 | | | 55 | 63 | 48 | 29.76 | 29.71 | 95 | 85 | SSW | S | Fair | — | — |
| 18 | | | 53 | 63 | 55 | 29.65 | 29.65 | 98 | 97 | SE | S | Foggy | — | Foggy |
| 19 | | | 56 | 62 | 54 | 29.64 | 29.66 | 98 | 93 | SSE | WSW | — | Rain | Rain |

The quantity of Rain fallen in the month of September, was 2 inches and 75.100ths.

